# A study of a population of *Nyssomyia trapidoi* (Diptera: Psychodidae) caught on the Pacific coast of Ecuador

**DOI:** 10.1186/1756-3305-5-144

**Published:** 2012-07-23

**Authors:** Zapata S, Mejía L, Le Pont F, León R, Pesson B, Ravel C, Bichaud L, Charrel R, Cruaud C, Trueba G, Depaquit J

**Affiliations:** 1 Université de Reims Champagne-Ardenne, ANSES, EA 4688 USC Transmission vectorielle et épidémiosurveillance de maladies parasitaires, VECPAR, France; 2 Instituto de Microbiología, Universidad San Francisco de Quito, Quito, Ecuador; 3 72 Rue de la Colonie, Paris, France; 4 Faculté de Pharmacie, Université de Strasbourg, Strasbourg, France; 5 French Reference Centre on Leishmaniasis, University of Montpellier, UMR5290, 39 Av. Charles Flahault, F34295, Montpellier, France; 6 Aix Marseille Univ, IRD French Institute of Research for Development, EHESP French School of Public Health, UMR_D 190 "Emergence des Pathologies Virales, and IHU Mediterranee Infection, APHM Public Hospitals of Marseille, 13005, Marseille, France; 7 Genoscope, Centre National de Séquençage, 2 rue Gaston Crémieux, CP5706, 91057 Evry, Cedex, France

**Keywords:** Cryptic species, Isoenzymes, Mitochondrial DNA, Sympatry, *Endotrypanum*, Phlebovirus

## Abstract

**Background:**

Cutaneous leishmaniasis is endemic to the Pacific coast of Ecuador, and *Nyssomyia trapidoi* is considered to be its main vector. Dujardin *et al.* [1] recorded some differences in body pigmentation and isoenzymatic profiles in sympatric populations of *Ny. trapidoi* from the Pacific coast of Ecuador and suggested the existence of two cryptic species.

**Methods:**

Entomological collections were performed in November 2008 and March 2011 in the locality of Paraíso Escondido using CDC miniature light traps and human bait. Morphological, isoenzymatical and molecular (sequencing of cytochome *b* and cytochrome *c* oxidase 1 of the mitochondrial DNA) analyses, such as detection of *Leishmania* DNA and phlebovirus RNA in some females, were performed.

**Results:**

Neighbor-joining trees from mitochondrial sequences grouped all of Ecuadorian *Ny. trapidoi* (including the two color variants) in one cluster, except for two specimens which clustered separately in both genes. Isoenzymatic characterization confirmed that the color variants belong to the same population. Additionally, 11.5% of females were found by PCR to contain *Endotrypanum monterogeii* kinetoplastid DNA. All pools of *Ny. trapidoi* were negative for phlebovirus RNA.

**Conclusion:**

Analysis of mitochondrial gene sequences and isoenzymes was unable to support the existence of two sibling species within *Ny. trapidoi*, which is a probable vector of *Endotrypanum monterogeii.*

## Background

Currently, 76 species of phlebotomine sand flies belonging to 17 genera have been reported for Ecuador [2]; of these, *Nyssomyia trapidoi, Lutzomyia hartmanni, Lutzomyia gomezi* and *Lutzomyia ayacuchensis* are proven vectors of *Leishmania* parasites [3]. Leishmaniasis was first reported in Ecuador in 1920 [4] and is now endemic in the coastal region. It is present in 22 of the country’s 24 provinces and purportedly has an incidence of approximately 1,650 cases per year [5] (likely unknown) in various clinical forms: cutaneous (CL), mucocutaneous (MCL), diffuse cutaneous (DCL) and recidiva cutis (LRC) [3,6,7].

In the Pacific region of Ecuador, the sand fly species *Nyssomyia trapidoi* is distributed throughout the western foothills of the Andes and along the coast and is an important vector of *Leishmania (Viannia) panamensis*[3,8-10]. *Ny. trapidoi* is highly anthropophilic [11-13] and is commonly collected in secondary forests, crop plantations and close to human dwellings. The same features were observed in the original description of *Ny. trapidoi* by Fairchild & Hertig [14] who analyzed specimens from Panama (type-locality) and from the Ecuadorian Coast. A recent study in Panama found a high prevalence (43.3%) of *Leishmania naiffi* in *Ny. trapidoi*[15].

In 1996, an entomological collection carried out in two communities on the north coast of Ecuador, obtained specimens of *Ny. trapidoi* showing a slight color variation. Additionally, isoenzymatic analysis revealed a possible presence of two species living in sympatry [1], although they did not correspond to the color variants. The purpose of the present study was to confirm the existence of two species under the name *Ny. trapidoi* at these locations. This report describes the results of an isoenzymatic and mitochondrial DNA analysis carried out on specimens of *Ny. trapidoi* collected in 2008 and 2011 at one of the locations described previously by Dujardin *et al.*[1]. Moreover, we investigated the vectorial role of *Ny. trapidoi* for Trypanosomatids and phleboviruses.

## Methods

### Sand fly collection

Sand flies were captured in November 2008 and March 2011 in the locality of Paraíso Escondido (00° 85' 03" N, 79° 17' 49" W), Pichincha Province, using CDC miniature light traps. Moreover, female sand flies were collected manually on human bait (captured on the skin of the authors). An out-group was analyzed consisting of specimens from Nicaragua (Musun), a place close to the type-locality of the species. Ecuadorian specimens were identified based on color phenotypes: light (B), dark (G) and indistinguishable (T). Specimens were killed using carbon dioxide and immediately stored in 96% ethanol for molecular studies and liquid nitrogen for isoenzymatic analysis. Once in the laboratory, the thorax of each specimen was separated and stored at −20°C for subsequent DNA extraction. The specimens used for isoenzymatic study and virus detection were processed as described below. The head, wings and genitalia of each specimen processed for morphology, molecular biology or isoenzyme analysis were cleared in boiling Marc-André [16] solution and mounted between slide and cover slide. The specimens selected for virus detection and isolation were individually identified in a drop of sterile saline solution under a stereomicroscope and pooled in groups of 50 belonging to the same species and of the same genus. The specimens we were unable to identify according to the latter method were stored at 80°C for future studies.

### Isoenzyme analysis

Isoelectrofocusing was carried out in ultrathin agarose gels (Multiphor^TM^ II Electrophoresis system, GE Healthcare Life Sciences) with the ampholyte at pH 4.0-6.5 in accordance with the protocols described by Pesson *et al.*[17]. The following isoenzyme systems were tested: malate dehydrogenase (MDH, E.C.1.1.1.37), isocitrate dehydrogenase (ICD, E.C.1.1.1.42), glycerol-3-phosphate dehydrogenase (αGPDH, E.C.1.1.1.8), glucose-6-phosphate dehydrogenase (6PGD, E.C.1.1.1.44), hexokinase (HK, E.C.2.7.1.1), phosphoglucomutase (PGM, E.C.5.4.2.2), fumarase (FUM, E.C.4.2.1.2), and glucose phosphate isomerase, (GPI, E.C.5.3.1.9). The alleles for each locus were visualized as colored bands on the gels and numbered from the lowest to the highest pHi.

Allele frequencies, tests for deviation from Hardy-Weinberg equilibrium and Nei’s [18] genetic distance between the two groups of sand flies were calculated using BIOSYS-2 [19]. Genotypic linkage disequilibrium and genotypic differentiation were tested using GENEPOP 3.3 [20].

### Molecular taxonomy of phlebotomine sand flies

DNA was extracted using the QIAmp®DNA Mini Kit (Qiagen, Germany) and following the protocol used by Depaquit *et al.*[21]. Polymerase chain reactions (PCR) were performed for 30 *Ny. trapidoi* specimens. Each PCR was carried out in a 50 μl volume using 5 μl of DNA extracted solution and 50 pmol of the primers LepF and LepR [22] and C3B-PDR / NIN-PDR [23], as described previously, to amplify, respectively, cytochrome *c* oxidase 1 (COI) and cytochrome *b* genes from sand fly mitochondrial DNA. Amplification conditions for COI were as follows: an initial denaturation step at 94°C for 3 min followed by 5 cycles of 94°C denaturation for 30 s, 45°C annealing for 90 s, and 68°C extension for 60 s followed by 35 cycles of 94°C denaturation for 30 s, 51°C annealing for 30 s, 68°C extension for 60 s and, finally, a 68°C extension for 10 min [24,25]. For cytochrome *b*, an initial denaturation step at 94°C for 3 min was followed by 5 cycles (94°C for 30 s, 40°C for 30 s and 68°C for 60 s) then 35 cycles (94°C for 30 s, 44°C for 30 s and 68°C for 60 s), with a final extension at 68°C for 10 min [23].

PCR products were analyzed by electrophoresis in 1.5% agarose gels stained with 0.1% ethidium bromide. Amplicons were sequenced in both directions using the primers for DNA amplification.

DNA sequences were edited using the Pregap and Gap programs from the Staden Package software [26]. Sequence alignments were done using ClustalW software [27] and checked by eye. We selected a Neighbor-joining (NJ) analysis, which is a suitable method for intraspecific characterization of populations [28,29]. NJ was performed using MEGA 4.0 software [30]. Node support carried out using the NJ method was assessed by bootstrapping with 500 replications.

### Trypanosomatid DNA detection

Detection of trypanosomatid DNA was carried out by PCR using two segments: a 120 bp kDNA corresponding to the conserved region of kinetoplast minicircles (primers JW11, 5’-CCT ATT TTA CAC CAA CCC CCA GT-3’ and JW12, 5’-GGG TAG GGG CGT TCT GCG AAA-3’) [31] and the ribosomal small sub-units (SSU) (primers: KRD5, 5'-GATCTGGTTGATTCTGCCAGTAG-3' and KRD3, 5'-GATCCAGCTGCAGGTTCACCTAC-3') [32]. As positive controls, DNA samples from *Leishmania* reference strains belonging to *L*. *panamensis, L. guyanensis, L. braziliensis* and *L. peruviana* were used. The conditions for these PCR reactions were the same as those described by Nicolas *et al.*[31] and Clark *et al.*[32].

### Detection of phleboviruses in sandfly pools by RT-PCR

Twelve pools, each containing 50 *Ny. trapidoi* sand flies (a total of 550 females and 50 males) were ground using a Mixer Mill MM300 (Qiagen) with a 3-mm tungsten bead at a frequency of 30 cycles s^−1^ for 3 min in the presence of 600 μl Eagle's minimal essential medium supplemented with 5% decomplemented calf serum, 1% L-Glutamine and 100 IU penicillin G ml^−1^, 100 mg kanamycin ml^−1^, 100 mg streptomycin ml^−1^ and 7.5 μg amphotericin B ml^−1^. The resulting mixture was clarified by centrifugation at 5800 g for 10 min and the supernatant fluid aliquoted (three aliquots of 200 μl for each pool) and stored at −80°C. One 200 μl aliquot was used for viral DNA and RNA purification using a BioRobot EZ1XL and the EZ1 Virus Mini kit (Qiagen), eluted into 90 μl and stored at −80°C. 10 μl was subsequently used in each RT-PCR reaction. Two sets of primers targeting different genes were used in independent reactions: (i) phlebovirus consensus primers targeting the polymerase gene in the L RNA segment [33], (ii) primers specific for the nucleoprotein gene in the S RNA segment of phleboviruses within the Sandfly fever Naples virus complex [34]. The RT-PCR cycling conditions consisted of 48°C for 45 min and 94°C for 2 min, followed by 40 cycles at 94°C for 30 s, 45°C for 1 min and 68°C for 45 s, with a final elongation step at 68°C for 7 min. Nested PCRs were performed using the same conditions with 1.25 U Taq DNA polymerase (Invitrogen, USA). PCR products were visualized in a 2 % TAE/agarose electrophoresis gel and sequenced in both directions either directly or after cloning in a TA cloning vector.

### Virus isolation

Pooled sandfly homogenates stored at −80°C were used to inoculate Vero cells. Briefly, 100 μl each homogenate was diluted with 900 μl Eagle's minimal essential medium without fetal bovine serum (FBS), but enriched with antibiotics (100 IU penicillin G ml^−1^, 100 mg streptomycin ml^−1^, 100 mg kanamycin ml^−1^ and 7.5 μg amphotericin B ml^−1^) and used to seed Vero monolayers in a 12.5-cm^2^ flask. After incubation at room temperature for 1 hr, 4 ml of fresh 5% FBS medium was added. The flask was incubated at 37°C in an atmosphere containing 5% CO_2_. Flasks were examined daily for the presence of a cytopathic effect, and 400 μl supernatant was removed and tested by RT-PCR after viral RNA/DNA extraction as aforementioned.

## Results and Discussion

Entomological captures using human baits resulted in 93% *Ny. trapidoi,* 4.2% *Psychodopygus panamensis* and 2.8% *Lutzomyia* (*Helcocyrtomyia*) *hartmanni.* Captures with CDC light traps yielded, in order of abundance: *Ny. trapidoi*, *Trichophoromyia reburra, Pressatia dysponeta, Psathyromyia aragoi, Psychodopygus panamensis, Lutzomyia hartmanni, Psathyromyia abunaensis* and *Psychodopygus carrerai thula* respectively (Table 1). Sixty-four *Ny. trapidoi* females were used for isoenzyme analysis and 550 *Ny. trapidoi* females and 50 *Ny. trapidoi* males were processed for virus detection. Of the males captured with CDC light traps, a small proportion was subsequently studied. They belonged to the same species as the females and most were *Ny. trapidoi*. They were stored at −80°C.

**Table 1:** Phlebotomine sand fly species collected using two methods of capture

**Genus**	**Species**	**Method of capture**	**Sex**	**Number of specimens**	**Percentage (%)**
*Nyssomyia*	*trapidoi*	Human baits	♀	266	93
*Psychodopygus*	*panamensis*	Human baits	♀	12	4.2
*Helcocyrtomyia*	*hartmanni*	Human baits	♀	8	2.8
*Nyssomyia*	*trapidoi*	CDC	♀	108	38.7
*Trichophoromyia*	*reburra*	CDC	♀	102	36.6
*Pressatia*	*dysponeta*	CDC	♀	18	6.5
*Psathyromyia*	*aragoi*	CDC	♀	15	5.4
*Psychodopygus*	*panamensis*	CDC	♀	12	4.3
*Helcocyrtomyia*	*hartmanni*	CDC	♀	12	4.3
*Psathyromyia*	*abunaensis*	CDC	♀	6	2.2
*Psychodopygus*	*carrerai thula*	CDC	♀	6	2.2

### Analysis of sand fly mitochondrial DNA

**Figure 1:**
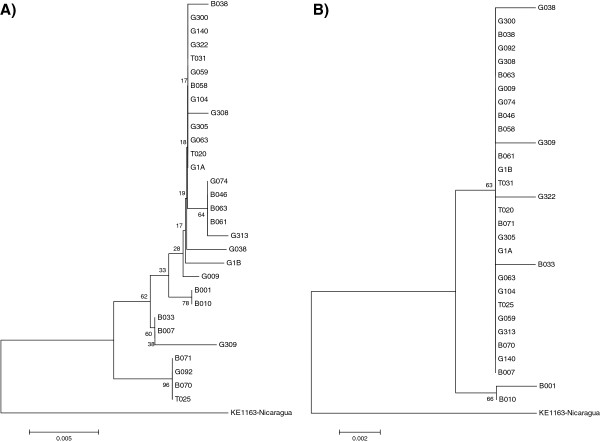
**Neighbor joining trees obtained from nucleotide analysis of: A) COI mtDNA and B) cytochrome*b*mtDNA sequences of the 30 Ecuadorian specimens of*Nyssomyia trapidoi*and one from Nicaragua.** Bootstrap values are shown in nodes (500 replicates).

Mitochondrial COI and cytochrome *b* sequences from 30 *Ny. trapidoi* females were obtained and submitted to Genbank (accession numbers JQ 322908 trough JQ 322968). Nucleotide sequences from COI amplicons (683 bp) revealed 31 variable sites, 10 of which were informative; cytochrome *b* (511 bp) contained 16 variable sites, two of them informative. The nucleotide composition was 37% T, 19% C, 28% A and 17% G for COI and 38% T, 15% C, 39% A and 9% G for cytochrome *b*. The alignment showed no indel in either sequence. COI sequences showed an average base substitution per site of 0.6% and cytochrome *b* sequences showed an average of 0.25%*.* There are more COI haplotypes than cyt b haplotypes. (Figure 1).

### Analysis of isoenzyme polymorphism

Since MDH and ICD each showed two distinct loci, a total of 10 loci were studied. Six of these gave clearly interpretable polymorphic patterns: GPI, PGM, HK, FUM, 6PGD and MDH1. Samples tested for αGPDH were monomorphic. Table 2 shows the number of sand flies and allelic frequencies at each polymorphic locus.

**Table 2:** **Allelic frequencies at the six polymorphic isoenzyme loci characterized in light and dark populations of*Nyssomyia trapidoi*. n : sample size;*P*: probability of χ**^**2**^**value occuring by chance, when testing for deviation from Hardy-Weinberg expectations of genotypes frequencies**

**Locus**	**Populations**	
	***Nyssomyia trapidoi*LIGHT**	***Nyssomyia trapidoi*DARK**
PGI		
n	33	31
1	0.879	0.806
2	0.076	0.065
3	0.045	0.113
4	0.000	0.016
*P*	*1.000*	*0.558*
PGM		
n	33	32
1	0.803	0.891
2	0.015	0.000
3	0.182	0.109
*P*	*0.077*	*0.306*
HK	33	31
1	0.955	1.000
2	0.015	0.000
3	0.030	0.000
*P*	*1.000*	-
FUM		
n	22	23
1	0.932	1.000
2	0.068	0.000
*P*	*1.000*	-
6 PGD		
n	22	23
1	0.932	0.848
2	0.023	0.022
3	0.023	0.109
4	0.023	0.022
*P*	*1.000*	*1.000*
MDH 2		
n	21	22
1	0.976	1.000
2	0.024	0.000
*P*	*1.000*	-

n : sample size; P: probability of χ2value occuring by chance, when testing for deviation from Hardy-Weinberg expectations of genotypes frequencies.

Both populations of light and dark sand flies were in Hardy-Weinberg equilibrium (P > 0.05). When combined as one group, only PGM deviated (P = 0.033); this disequilibrium was due to a deficiency of heterozygotes. There was no significant linkage disequilibrium for either populations tested alone or grouped together (1 > P >0.180). Nei’s genetic distance value was 0.004 and there was no genotypic differentiation between light and dark *Ny. trapidoi* ( χ^2^ = 12.309, P = 0.421).

### Detection of trypanosomatid DNA

Out of 78 *Ny. trapidoi* females tested by PCR for the presence of trypanosomatid kDNA, 9 (11.5%) were positive. BLAST analysis of the 120 bp PCR products failed to reveal any significant score. However, 8 out of 78 kDNA PCR positive females were also PCR positive for the SSU rDNA locus. All the 2,135 bp sequences obtained from our samples were similar and showed 100% identity to the *Endotrypanum monterogeii* sequence (X53911) described by Fernandes *et al.*[35]. One sequence was deposited in Genbank (JQ863389).

### Detection of phleboviruses in sandfly pools by RT-PCR and by viral isolation

#### No pools were positive for phleboviruses using RT-PCR or by isolation on vero cells

## Conclusion

The results of this study confirmed that *Ny. trapidoi* is the most common species captured [9] in the Paraíso Escondido community in the central coastal region of Ecuador. Two other anthropophilic species, *Psychodopygus panamensis* and *Lutzomyia hartmanni*, were recorded at this locality. Other non-anthropophilic species, i.e. *Trichophoromyia reburra, Pressatia dysponeta, Psathyromyia aragoi, Lutzomyia hartmanni, Psathyromyia abunaensis* and *Psychodopygus carrerai thula* were also collected there (Table 1).

**Figure 2:**
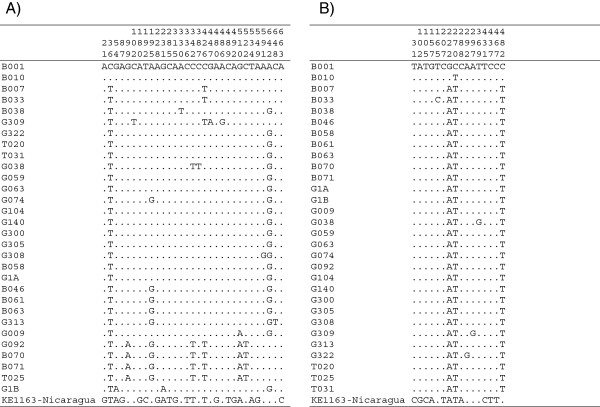
**Variable nucleotides found in DNA sequence alignments.A)** COI mt DNA (683 bp) and** B)** cytochrome *b* mtDNA (511 bp). Identical bases are represented by dots. Numbers above indicate the positions in the nucleotide sequence.

Molecular (mtDNA sequencing) and isoenzymatic analyses did not support the existence of two populations (possible cryptic species) at Paraíso Escondido as previously hypothesized by Dujardin *et al.*[1]. The topology of the NJ tree obtained using cytochrome *b* sequences (Figure 1) grouped all Ecuadorian *Ny. trapidoi* into two clusters. The first cluster comprised the specimens B001 and B010 and the second cluster grouped the other specimens. This dichotomy is supported by only two nucleotide substitutions (positions 222 and 482). The *Ny. trapidoi* from Nicaragua differed from the Ecuadorian specimens in 12 nucleotide positions (Figure 2).

On the other hand, the NJ tree of COI sequences showed 5 clusters (Figure 1). The main branch included many of the specimens sharing the main cytochrome *b* haplotype. Another branch includes the B001 and B010 specimens, which grouped exactly as in the cytochrome *b* NJ clustering, thus perhaps warranting additional studies. These two specimens differ from the main haplotype by two nucleotide positions: 35 and 642. Specimens B007, B033 and G309 make up a third cluster that is supported mainly by one mutation. The fourth cluster (specimens B070, B071, G092 and T025) is strongly supported by seven positions (Figure 2). It is interesting to note that there is no correlation between haplotypes and color phenotypes. In this case, the COI gene provided a greater level of stratification than the cytochrome *b* gene.

Isozyme analysis suggested that the two color variants are conspecific *Ny. trapidoi*, and we did not find any variation of αGPDH profiles in our samples. However, the electrophoresis protocol used by Dujardin *et al.*[1] was different from that used in this study.

*Ny. trapidoi* is known to be an efficient vector for the vesicular stomatitis virus (Vesiculovirus) [36,37]. According to our study, there is no evidence for the circulation of any phlebovirus in *Ny. trapidoi* in the locality of Paraíso Escondido.

In this work, we processed 78 *Ny. trapidoi* females, 2 engorged on human blood and the rest unfed. Nine (11.5%) of the unfed sand flies had identical trypanosomatid kDNA sequences and showed no homology to *Leishmania panamensis* and *L. guyanensis*, species commonly found in *N. trapidoi*[3]. However, SSU rDNA sequences (2135 bp) from the same specimens showed 100% homology with the *Endotrypanum monterogeii* sequence (GenBank X53911) obtained from a sloth (*Choloepus hoffmanni)* and described by Fernandes *et al.*[35]. Moreover, our sequences showed a high homology (over more than 2100 bp) to sequences described as *Endotrypanum* sp. by Ferreira *et al.*[38]: 99.7%, homology to *Endotrypanum* isolated from *Lutzomyia gomezi* (EU21238) and 99.8-99.9% to isolates from *Psathyromyia dendrophyla* (EU21239, EU21240). If we consider this variability as intraspecific, it might suggest that *E. monterogeii* has a wide spectrum of vectors, including the genera *LutzomyiaPsathyromyia* and *Nyssomyia*. Although sloths were common in study sites some time ago, they are now rare, and other vertebrates may play a role as reservoirs. Further studies are needed to learn more about the vertebrate hosts of *E. monterrogeii*, since to date our knowledge is limited to *C. hoffmanni*[35,39].

## Competing interests

The authors declare that they have no competing interests.

## Authors’ contributions

SZ, FLP, RL & JD were responsible for the conception and design of the work and have been involved in drafting the manuscript. SZ & BP carried out the isoenzymatic analysis and have given final approval of the version to be published. SZ, JD and CR did the parasitologic study and have given final approval of the version to be published. CC did the sequencing of the samples through the project @speed-ID and have given final approval of the version to be published. LM & GT helped with collecting and processing the samples, revising the manuscript critically and have given final approval of the version to be published. LB and RC carried out the viral study, critically reviewed the manuscript and have given final approval of this version to be submitted for publication.
